# The efficacy of 2-nitrovinylfuran derivatives against *Leishmania* in vitro and in vivo

**DOI:** 10.1590/0074-02760140324

**Published:** 2015-04

**Authors:** Sergio Sifontes-Rodríguez, Lianet Monzote-Fidalgo, Nilo Castañedo-Cancio, Ana Margarita Montalvo-Álvarez, Yamilé López-Hernández, Niurka Mollineda Diogo, Juan Francisco Infante-Bourzac, Oliver Pérez-Martín, Alfredo Meneses-Marcel, José Antonio Escario García-Trevijano, Miguel Ángel Cabrera-Pérez

**Affiliations:** 1 Centro de Bioactivos Químicos, Universidad Central Martha Abreu de Las Villas, Santa Clara, Villa Clara, Cuba; 2 Instituto de Medicina Tropical Pedro Kourí, La Habana, Cuba; 3 Centro de Biociencias, Universidad Autónoma de San Luis Potosí, San Luis Potosí, SLP, México; 4 Instituto Finlay, La Habana, Cuba; 5 Departamento de Parasitología, Facultad de Farmacia, Universidad Complutense de Madrid, Madrid, España

**Keywords:** nitrovinylfuran, furvina, UC245, Leishmania, BALB/c, promastigote

## Abstract

Despite recent advances in the treatment of some forms of leishmaniasis, the available drugs are still far from ideal due to inefficacy, parasite resistance, toxicity and cost. The wide-spectrum antimicrobial activity of 2-nitrovinylfuran compounds has been described, as has their activity against Trichomonas vaginalis and other protozoa. Thus, the aim of this study was to test the antileishmanial activities of six 2-nitrovinylfurans in vitro and in a murine model of leishmaniasis. Minimum parasiticide concentration (MPC) and 50% inhibitory concentration (IC_50_) values for these compounds against the promastigotes of Leishmania amazonensis, Leishmania infantum and Leishmania braziliensis were determined, as were the efficacies of two selected compounds in an experimental model of cutaneous leishmaniasis (CL) caused by L. amazonensis in BALB/c mice. All of the compounds were active against the promastigotes of the three Leishmania species tested. IC_50_ and MPC values were in the ranges of 0.8-4.7 µM and 1.7-32 µM, respectively. The compounds 2-bromo-5-(2-bromo-2-nitrovinyl)-furan (furvina) and 2-bromo-5-(2-methyl-2-nitrovinyl)-furan (UC245) also reduced lesion growth in vivo at a magnitude comparable to or higher than that achieved by amphotericin B treatment. The results demonstrate the potential of this class of compounds as antileishmanial agents and support the clinical testing of Dermofural^(r)^ (a furvina-containing antifungal ointment) for the treatment of CL.

Leishmaniasis remains one of the most neglected diseases worldwide; 350 million people are considered at risk of contracting leishmaniasis and some two million new cases occur each year. In the past 10 years, major scientific breakthroughs have been made in the treatment, diagnosis and prevention of leishmaniasis and the prices of several key medicines have been reduced. However, mortality and morbidity from leishmaniasis worldwide show a worrisome increasing trend (WHO 2010 ). There is a pressing need to replace the few drugs that are currently available and that have substantial limitations in terms of tolerability, efficacy, cost and parasite resistance. Encouraged approaches include repurposing existing drugs, exploring drug combinations to avoid resistance and searching for new drugs (WHO 2012).

The antifungal and antibacterial activities of nitrovinyl (nitroethenyl) compounds of benzene, thiophene and furan have long been demonstrated (Bellenghi & Wittgens 1958, Alabi & Owolabi 2012, Scholz et al. 2013). Although the presence of a nitro group in position 5 of the furan ring is not indispensable for the biological activity of 2-vinylfurans (Drobnica & Sturdik 1980), it does determine their genotoxic potential (Estrada 1998). Thus, a clear distinction between nitrofurans (nitrogen bonded to the furan ring) and nitrovinylfurans (nitrogen bonded to the side vinylic chain) should be made (Fig. 1).

**Fig. 1:**
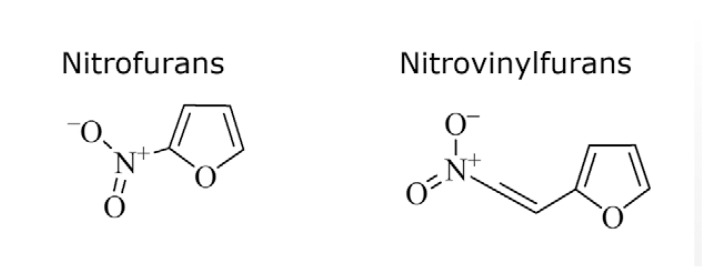
structural difference between nitrofurans (nitrogen bonded to the furan ring) and nitrovinylfurans (nitrogen bonded to the side vinylic chain).

Well-known nitrofurans such as nitrofurazone, nitrofurantoin and nitrofurazolidone that have been used as antibacterial drugs for years have been removed from the pharmaceutical market in many countries as a consequence of their mutagenic potential (Snyder & Green 2001). Conversely, in vivo genetic toxicity tests conducted on nitrovinylfurans have failed to demonstrate a genotoxic potential (Borroto et al. 2005).

The present paper reports testing of the antileishmanial activities of six 2-nitrovinylfurans that have previously been demonstrated to have activity against bacteria, fungi (Blondeau et al. 1999) and protozoan parasites such as *Eimeria *sp. (González-Díaz et al. 2007) and *Trichomonas vaginalis *(Meneses-Marcel et al. 2008).

## MATERIALS AND METHODS

*Parasites and cultures* - Reference strains of *Leishmania amazonensis *(MHOM/77/LTB0016), *Leishmania infantum* (MHOM/FR/78/LEM75) and *Leishmania braziliensis* (MHOM/BR/75/M2 903) were used. Parasites were cultivated in Schneider's Insect Medium (Sigma-Aldrich, USA) supplemented with 10% heat inactivated (56ºC, 30 min) foetal bovine serum (HyClone^(r)^, USA) and incubated at 26ºC. Parasites were kept in the exponential multiplication phase by passaging every three-four days.

*Growth curves* - The following procedure was performed for the three *Leishmania* species used in the experiment. The first six columns of a 96-well culture plate were seeded with 200 µL of log-phase parasite suspensions containing 5 x 10^5^ promastigotes/mL. A similar volume of culture medium (without parasites) was added to the rest of the plate. Immediately after seeding the plate and every 24 h, 5 µL of 2 µg/mL amphotericin B (AmPB) (Fungizone(tm), Bristol Myers Squibb, France) was added to one row of the plate. This procedure allowed for cultures to be inactivated every 24 h from day 0-7. After seven days of incubation at 26ºC, 20 µL of 20 mg/mL p-nitro-phenyl-phosphate (Sigma-Aldrich) in ammonium acetate (pH 5.5) - 1% Triton X100 was added to each well. Plates were incubated for 3 h at 37ºC and absorbance was read in a Tecan Infinite 200 Pro microplate reader at 405 nm. The net absorbance was calculated subtracting the average value of the six wells on the right-hand side of each row to their counterparts on the left-hand side. The net absorbance was plotted against the incubation time to obtain growth curves for each strain.

*Test compounds* - Nitrovinylfuran derivatives were synthesised as previously described (Castañedo & Gaitan 2003). They were dissolved in dimethylsulfoxide (DMSO) (Sigma-Aldrich) and two-fold serial dilutions in the range of 4 mg/mL to 3.9 µg/mL were performed. Details on the structure and identification of the test compounds are shown in Table I.

**TABLE I.** Influence of structural changes of 2-nitrovinylfurans on their electronic properties and their antileishmanial activity

Compound	Structure	Charge at C1	Charge at C2	C2-C1	Average IC_50 _ (µM)
G0	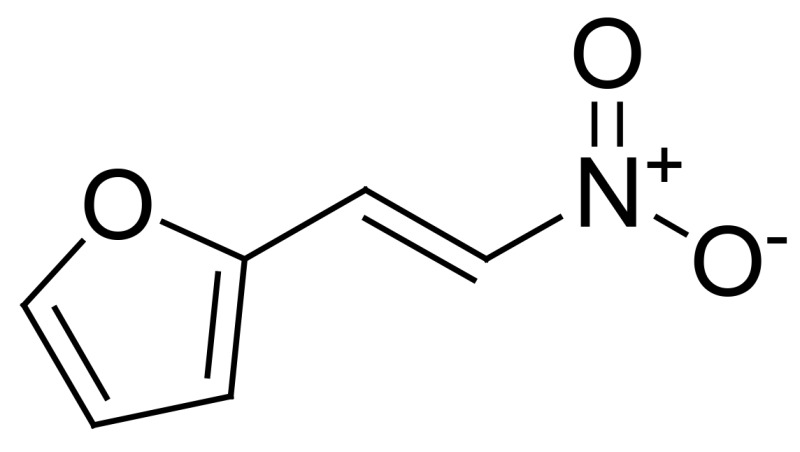	-0.104	0.053	0.157	2.870
MbA	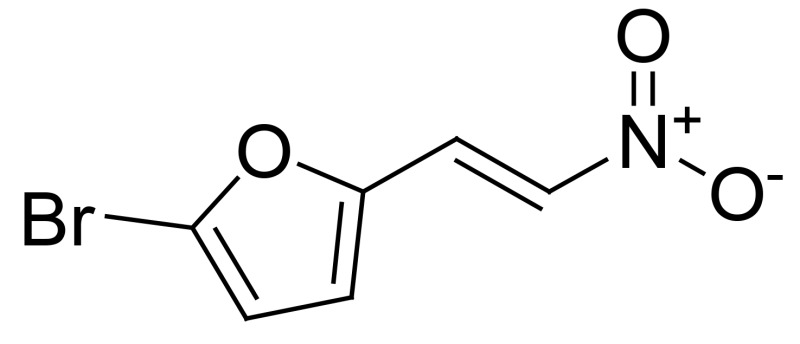	-0.108	0.053	0.161	1.330
MbC	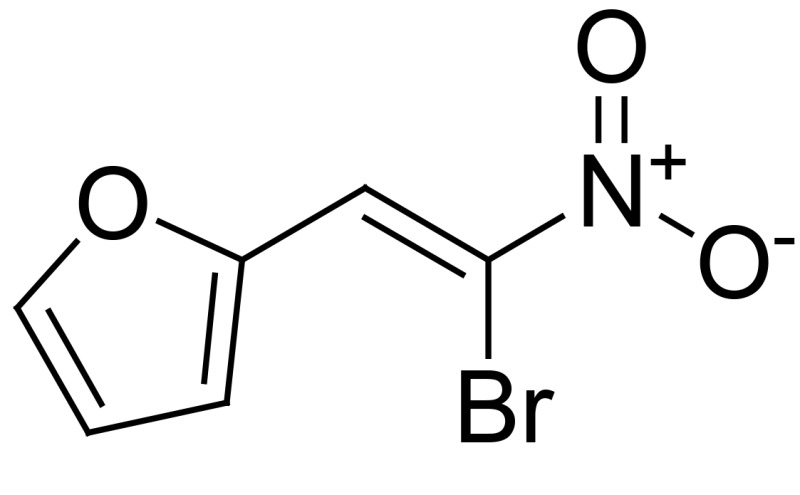	-0.113	0.170	0.283	1.900
Furvina	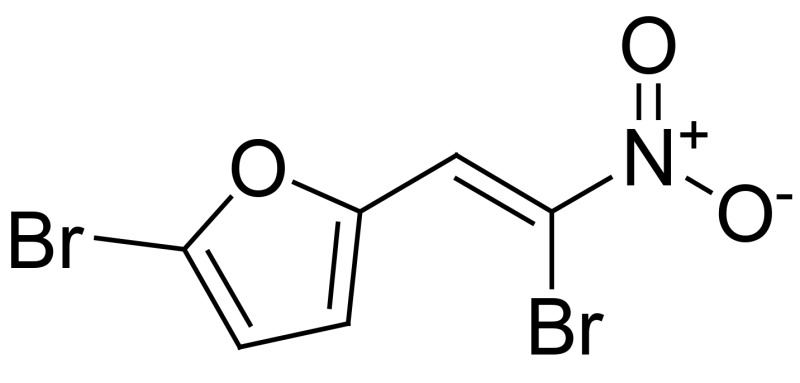	-0.117	0.169	0.286	0.970
UC244	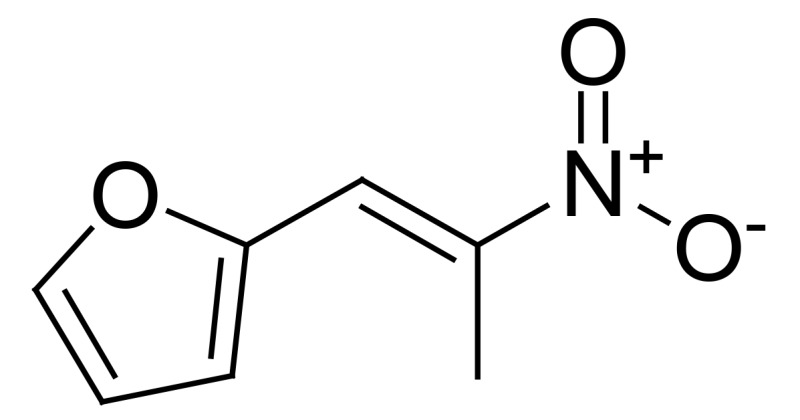	-0.146	0.226	0.372	2.170
UC245	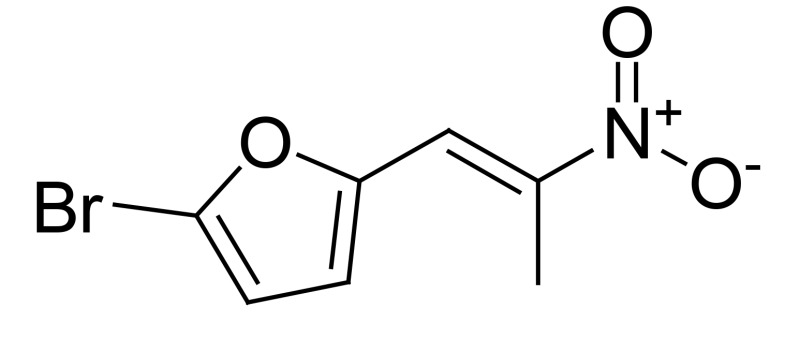	-0.151	0.226	0.377	0.930

density functional theory methods implemented in Gaussian 09 software were used to calculate Mulliken charges at atoms participating in the exocyclic double bond (charge 1 and charge 2), as well as the difference between them (C2-C1). Average 50% inhibitory concentration (IC50) are the arithmetic mean of IC50 values for the three species of Leishmania tested. Furvina: 2-bromo-5-(2-bromo-2-nitrovinyl)-furan; UC245: 2-bromo-5-(2-methyl-2-nitrovinyl)-furan.

*Inhibitory and parasiticide drug concentrations* - The concentration of each chemical capable of inhibiting parasite multiplication by 50% compared with the multiplication of nontreated cultures (IC_50_) and the concentration causing the total inhibition of parasite motility [minimum parasiticide concentration (MPC)] were used as drug efficacy indices. The IC_50_ was calculated as described elsewhere (Bodley et al. 1995). Briefly, the top half of a 96-well culture plate was seeded with 199 µL containing 5 x 10^5 ^promastigotes/mL and a similar volume of culture medium without parasites was added to the remainder of the plate. One drug concentration was assigned to each column of the plate and 1 µL of compound in DMSO was added per well. The highest concentration of DMSO was 0.5%, which is known to be harmless to parasite growth (Bodley et al. 1995). However, a number of control wells treated with 1 µL of DMSO were also included. After incubation for 72 h at 26ºC, the plate was observed under an inverted microscope and the MPC was calculated as the geometric mean (4 replicates) of concentrations that completely arrested promastigote motion. P-nitro-phenyl-phosphate was then added to the entire plate, which was incubated and read as previously described. The net absorbance corresponding to each drug concentration was calculated by subtracting the average absorbance of the four bottom wells (containing drug and culture medium) to that of the top wells (with drug, culture medium and an amount of parasites as a function of the drug concentration and its inhibitory effect). IC_50_ values were estimated by nonlinear fitting of the drug concentration vs. net absorbance curve to the Emax sigmoid model (Holford & Sheiner 1981). Each assay was repeated at least three times.

*Cytotoxicity against mammalian cells and selectivity index* - Cytotoxicity assays were conducted as described elsewhere (Lorente et al. 2004). Briefly, plates were seeded with 100 µL of HeLa-KB cells (subline of the keratin-forming tumour cell line HeLa, ATCC, CCL-17(tm)) at 4 x 10^4^/mL in RPMI-1640 plus 10% heat-inactivated foetal calf serum and incubated at 37ºC in 5% CO_2 _for 24 h. The overlay was removed and replaced by the test compounds in fresh medium at concentrations ranging from 30 µg/mL to 100 ng/mL in triplicate. The positive control drug was podophyllotoxin (Sigma-Aldrich). Plates were incubated for 72 h at 37ºC in 5% CO_2_; 10 µL of alamarBlue was then added to each well and the plates were incubated again for 4 h (37ºC, 5% CO_2_) before reading the fluorescence at EX/EM 530/585 nm (cut-off, 550 nm) in a Tecan Infinite 200 Pro microplate reader. The 50% cytotoxic concentrations (CC_50_) values were estimated by nonlinear fitting to the Emax sigmoid model (Holford & Sheiner 1981). Each assay was repeated at least three times. The selectivity index was calculated for each test compound as the CC_50 _in the mammalian cell line divided by the average of the IC_50_ values against the three *Leishmania* species. The existence of a significant correlation between the IC_50_ and CC_50_ values was assessed by nonparametric Spearman's rank order correlation analysis.

*Structure-activity relationship analysis* - Density functional theory methods were used to calculate Mulliken charges of the atoms for the test compounds using Gaussian 09 software (Frisch et al. 2009). The relationship between IC_50_ values and charge densities at carbon atoms participating in the exocyclic double bond as well as the difference of charges between them were analysed.

*In vivo assays* - Based on in vitro results and previously conducted toxicological tests, the compounds 2-bromo-5-(2-bromo-2-nitrovinyl)-furan (furvina) and 2-bromo-5-(2-methyl-2-nitrovinyl)-furan (UC245) were selected to assess their efficacy in a mouse model of experimental cutaneous leishmaniasis (CL). Two initial experiments were carried out in which treatment was begun as soon as lesions were evident; a third experiment was carried out using mice with chronic lesions.

*Animals* - Female, 16-18 g, six-eight weeks old BALB/c mice were supplied by the National Center for the Production of Laboratory Animals (Cuba). They were maintained under controlled environmental conditions (room temperature 22-25ºC, relative humidity 60-65%, light cycle 10 h light-14 h dark) and were handled by qualified personnel. At the end of the studies, the mice were sacrificed by an overdose of pentobarbital (100 mg/kg). The study protocol was approved by the Ethical Committee for the Care and Use of Laboratory Animals at Finlay Institute, which is in accordance with international standards on the topic.

*Experiment 1* - Female BALB/c mice were experimentally infected by intradermal injection in the footpads with 5 x 10^6^ stationary phase *L. amazonensis *promastigotes. Six weeks later, mice were randomised and then treated daily for 14 days by intraperitoneal injection with the test compounds. Furvina was administered at a dose of 5 mg/Kg, UC245 at 50 mg/Kg and AmPB at 1 mg/Kg. Miglyol 810 (Hüls AG, Germany), the vehicle used for the 2-nitrovinylfurans, was similarly administered to a group of infected mice. Another group of infected mice that did not receive any treatment at all was also included as control. The dorsoplantar thickness of the rear limbs was measured every week with a Vernier calliper and lesion sizes were calculated by subtracting the value of the noninfected limb (left) to that of the infected one (right). Groups were statistically compared by repeated measures analysis of variance and Fisher's least significant difference test using STATISTICA software (available from: statsoft.com ). p values under 0.05 were considered statistically significant.

*Experiment 2* - This test was performed in a similar manner as that described above. However, furvina was dosed at 2 mg/Kg. UC245 was dosed at 100 mg/Kg and treatments were administered every 12 h instead of every 24 h.

*Experiment 3* - Mice were similarly infected as described for the previous experiments. Treatments were also administered every 12 h, but were initiated at 18 weeks post-infection. AmPB was also used as a control, though at a dose of 5 mg/Kg given every other day.

## RESULTS

**Fig. 2:**
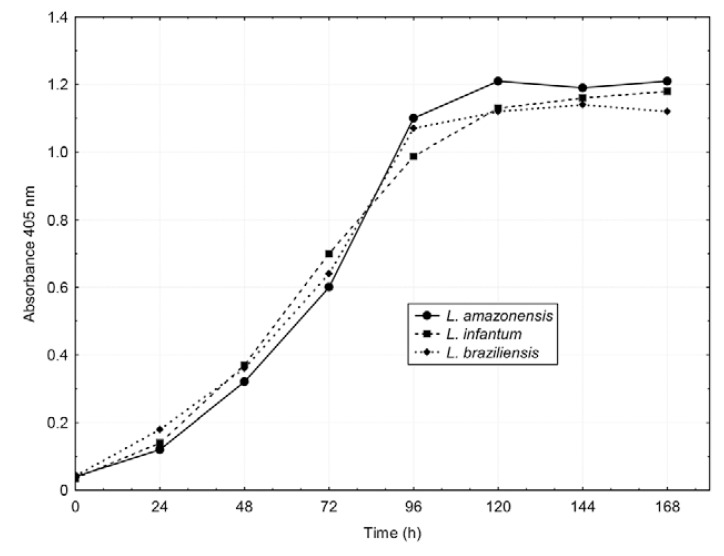
growth curves of Leishmania amazonensis, Leishmania infantum and Leishmania braziliensis in Schneider's medium. Exponentially growing promastigotes were seeded in 96-well plates at 5 x 105 promastigotes/mL. Every 24 h a group of wells was inactivated by addition of 5 μL, 2 μg/mL amphotericin B. After seven days of culture at 26ºC, cell density was estimated by measuring parasite phosphatases activity.

*Growth curves* - The three strains tested showed a similar growth pattern (Fig. 2), involving a short lag phase of less than 24 h, a three-day-long logarithmic phase from 24-96 h, a growth deceleration phase from 96-120 h and a stationary phase that lasted from 120 h to the seventh day of culture. Based on this result, a 72 h incubation period was set for drug screening assays with these three strains.

*In vitro activity and cytotoxicity* - All six compounds tested showed inhibitory effects against promastigotes in vitro (Table II). The susceptibilities of the three species to the test compounds were comparable (main effects ANOVA, p = 0.086) with IC_50_ values ranging from 0.18-0.66 µg/mL (0.82-4.7 µM). At higher concentrations (1.7-32 µM), the compounds were also lethal to the parasites; this was evident by the total immobilisation of promastigotes after 2-3 h of exposure. AmPB, used as positive control, showed IC_50_ values of 0.015-0.030 µM.

**TABLE II.** In vitro activity of 2-nitrovinylfuran derivatives against Leishmania promastigotes and cytotoxicity against KB cells

Compound	*Leishmania amazonensis*			*Leishmania braziliensis*			*Leishmania infantum*			KB cells	SI
	IC_50_	MPC		IC_50_	MPC		IC_50_	MPC		CC_50_	
G0	4.7 ± 0.4	8.1 ± 0.4		1.4 ± 0.3	9.3 ± 0.4		2.5 ± 0.4	12.6 ± 0.5		11.6 ± 3.6	4.0
MbA	2.1 ± 0.3	9.5 ± 0.4		0.8 ± 0.3	2.8 ± 0.4		1.1 ± 0.3	2.3 ± 0.5		6.0 ± 2.6	4.5
MbC	2.9 ± 0.3	8.1 ± 0.4		1.1 ± 0.4	5.7 ± 0.4		1.7 ± 0.2	9.2 ± 0.3		16.6 ± 3.8	8.7
Furvina	0.8 ± 0.3	5.2 ± 0.4		0.9 ± 0.4	1.7 ± 0.4		1.2 ± 0.3	2.9 ± 0.3		13.6 ± 2.2	14.1
UC244	2.2 ± 0.4	22.8 ± 0.9		2.3 ± 0.2	31.6 ± 0.5		2.0 ± 0.2	8.3 ± 0.5		25.4 ± 3.8	11.7
UC245	0.9 ± 0.2	6.1 ± 0.4		1.0 ± 0.3	5.7 ± 0.4		0.9 ± 0.3	2.7 ± 0.4		16.7 ± 1.8	17.9
Amphotericin B	29 ± 5	55 ± 7		14 ± 3	30 ± 6		14 ± 04	17 ± 5		-	-

nitrovinylfuran compounds dissolved in DMSO were added to exponentially growing promastigotes. After 72 h of incubation at 26ºC, minimal parasiticide concentrations (MPC) were determined by microscopic observation. Afterwards, parasite phosphatases activity was determined as a surrogate for parasite cell counts. Cytotoxicity was assessed in KB cells cultivated in 96 well plates at 36ºC and 5% CO2. Cell viability was estimated by reduction of alamarBlue 72 h after the addition of the test compounds. Fifty percent inhibitory concentrations (IC50) and 50% cytotoxic concentrations (CC50) were estimated by nonlinear fitting to the Emax sigmoid model. Results are the mean ± standard deviation of at least three representative assays conducted in quadruplicate. Concentrations are expressed in μM, but for amphotericin B which are in nm. Furvina: 2-bromo-5-(2-bromo-2-nitrovinyl)-furan; SI: selectivity index; UC245: 2-bromo-5-(2-methyl-2-nitrovinyl)-furan.

The electron density at C1 and thus the polarity of the double bond increased with the substitution of hydrogen by bromide in the furan ring (Table I) or by the presence of a methyl or bromide group at C2 of the vinylic chain; this increased polarity was associated with augmented antileishmanial activity. The paired comparison of compounds that differed in the presence of bromide in the furan ring (G0 vs. MbA, MbC vs. furvina and UC244 vs. UC245) showed a decreased Mulliken charge at C1 in the range of 0.004-0.005 that coincided with a two-fold increase in activity. Similarly, methylation (G0 vs. UC244, MbA vs. UC245) or bromination at C2 (G0 vs. MbC and MbA vs. furvina) decreased the Mulliken charge at C1 to 0.009 and 0.0047, respectively, and was also associated with increased antileishmanial activity.

KB cells were sensitive to podophyllotoxin, with a CC_50_ as low as 5.3 ± 1.4 nm. The CC_50 _values for the test compounds were in the range of 6-25 µM (Table II). There was no apparent correlation (Spearman's rank order correlation coefficient R = -0.086, p > 0.05) between the CC_50_ and IC_50_ values. The calculated selectivity indices based on the activity against promastigotes and cytotoxicity in KB cells were 4-18, with the highest values for UC245 and furvina. Thus, these two compounds were selected for activity assays against intracellular amastigotes. However, drug concentrations that were not toxic to mouse peritoneal macrophages used as host cells had an efficacy under 50% in reducing the percentage of infected cells (data not shown).

*In vivo efficacy assays* - Once the in vitro inhibitory and parasiticide activities of the test compounds against the promastigote stage had been demonstrated and after conducting preliminary toxicological experiments to find proper dosing schedules, a group of in vivo experiments was carried out to assess the efficacy of the compounds against experimental CL. Based on in vitro results and on the availability of chemical and pharmaceutical information, two compounds were selected for in vivo assays: furvina and UC245.

**Fig. 3:**
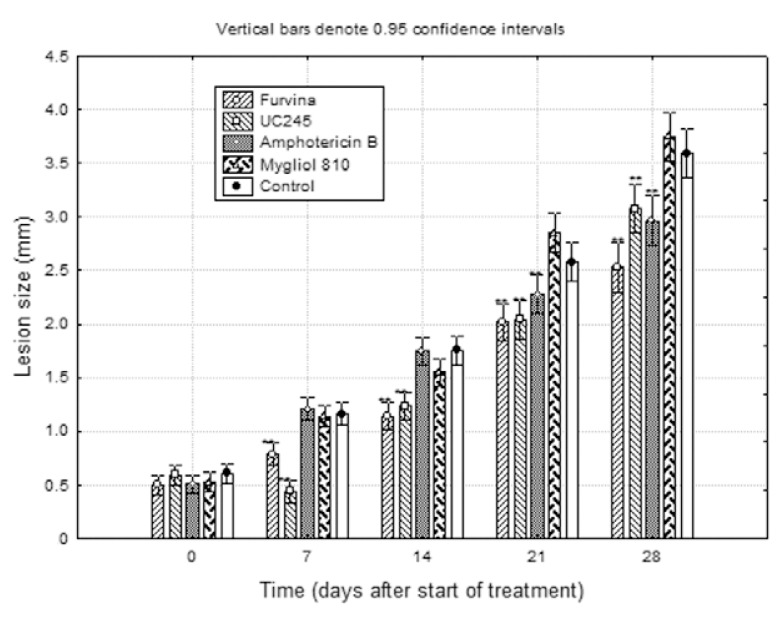
In vivo efficacy of 2-bromo-5-(2-bromo-2-nitrovinyl)-furan (furvina) and 2-bromo-5-(2-methyl-2-nitrovinyl)-furan (UC245) against experimental cutaneous leishmaniasis. Early stage lesions treated every 24 h. Mice were infected by intradermal injection with 5 x 106 stationary phase Leishmania amazonensis promastigotes. Six weeks later they were treated by intraperitoneal route every 24 h during 14 days with furvina (5 mg/Kg), UC245 (50 mg/Kg), amphotericin B (1 mg/Kg) or Miglyol 810 (0.1 mL). Control mice were infected, but received no treatment at all. Lesion size was measured every week using a Vernier caliper.

*Experiment 1* - Experimentally infected mice had evident lesions at six weeks post-infection. They were then treated once a day for 14 days with either furvina or UC245. The lesion sizes of mice treated with either of the test compounds were statistically smaller (p < 0.01) than those of the untreated control group during the four weeks of follow-up. Treated mice had minimal lesion growth during the first week (Fig. 3) and those treated with UC245 in particular showed a slight decrease. Similarly, mice treated with AmPB had statistically smaller lesions (p < 0.01) compared with the control group, but only during the two weeks after treatment had stopped. Mygliol-810, the solvent used for the nitrovinylfuran compounds, did not show any effect on the clinical course of the experimental infection; thus, a vehicle control group was no longer included in subsequent assays.

**Fig. 4:**
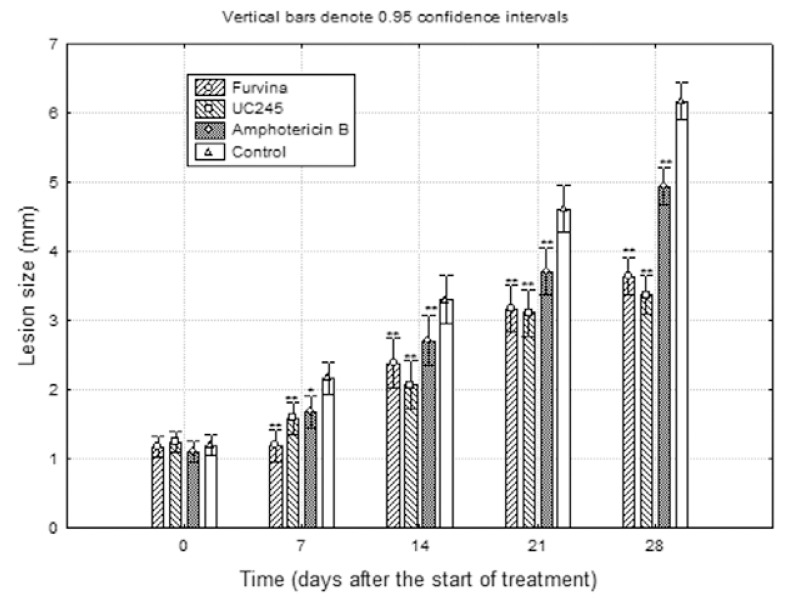
In vivo efficacy of 2-bromo-5-(2-bromo-2-nitrovinyl)-furan (furvina) and 2-bromo-5-(2-methyl-2-nitrovinyl)-furan (UC245) against experimental cutaneous leishmaniasis. Early stage lesions treated every 12 h. Mice were intradermally infected and treated by intraperitoneal route every 12 h during 14 days with furvina (2 mg/Kg), UC245 (100 mg/Kg) or amphotericin B (1 mg/Kg). Infected, nontreated control mice were also included. Once therapy started lesion size was measured every week for four weeks.

*Experiment 2* - Increasing the frequency of administration produced comparable results (Fig. 4) as those obtained in experiment 1 because both nitrovinylfuran compounds retarded lesion growth compared with the control untreated mice (p < 0.01). The compounds were similarly active (p > 0.05) and showed higher (p < 0.05) efficacies than that of AmPB. However, in this case, the magnitude of the difference with respect to the control group was higher.

**Fig. 5:**
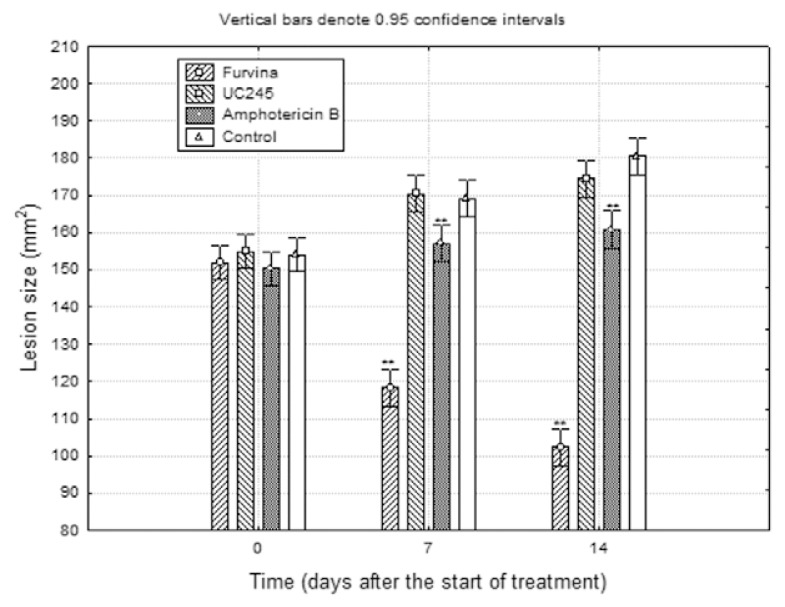
In vivo efficacy of 2-bromo-5-(2-bromo-2-nitrovinyl)-furan (furvina) and 2-bromo-5-(2-methyl-2-nitrovinyl)-furan (UC245) against chronic experimental cutaneous leishmaniasis. Eighteen weeks post-infection mice had developed lesions of 1.23 ± 0.24 x 1.24 ± 0.19 cm. Treatment was then initiated every 12 h for 14 days with furvina (2 mg/Kg), UC245 (100 mg/Kg) or amphotericin B (5 mg/Kg, every 48 h). The dorsoplantar and lateral diameters of lesions were measured and lesion size was estimated as their product.

*Experiment 3* - In the third experiment, the mice had developed chronic lesions at 18 weeks post-infection. The lesions were ulcerated and covered with a thick crust. The average dorsoplantar diameter of the infected foot was 1.23 ± 0.24 cm and the lateral diameter was 1.24 ± 0.19 cm. At this stage, treatment with either nitrovinylfuran derivatives or AmPB was started. Mice treated with furvina displayed progressively decreased lesion sizes, which significantly differed (p < 0.01) from the rest of the groups, including the AmPB-treated group. In contrast, UC245 did not show any efficacy in controlling the disease at this phase of development (Fig. 5).

## DISCUSSION

Nitrovinylfurans have activity against infectious bacteria, fungi and some protozoan parasites (Blondeau et al. 1999, Borroto et al. 2005, González-Díaz et al. 2007, Meneses-Marcel et al. 2008). Here, we demonstrated the antileishmanial activity of six 2-nitrovinilfuran derivatives against the promastigotes of representative species causing the CL (*L. amazonensis), *visceral* (L. infantum*) and mucocutaneous (*L. braziliensis)* forms of leishmaniasis, as well as their therapeutic efficacy in a model of CL caused by the experimental infection of BALB/c mice with *L. amazonensis*.

The growth kinetics of the parasites were studied before assessing the in vitro activity of the test compounds to assess extension of the log-phase to ensure that drug efficacy assays were conducted during that period. Although the beginning of the stationary phase was accurate, its end was not evident because a decline phase could not be demonstrated. When using p-nitro-phenyl-phosphate to estimate cell density, all of the parasites present, either living or dead, are similarly quantified (Bodley et al. 1995); consequently, it was not possible to visualise the point at which parasite death surpassed the multiplication rate, i.e., the decay phase. However, for the purpose of the present study, a proper log phase was demonstrated and an incubation time of 72 h could be established for the three strains to perform drug screening assays. We found this method to be practical, although it could be simpler if a colourless leishmanicidal product were used instead of AmPB, as there would be no need to include control wells to calculate the net absorbance values.

AmPB, the drug used as positive control for the in vitro and in vivo experiments, was active against the three species of *Leishmania* tested and its IC_50_ value was in the range reported elsewhere for this drug (Vermeersch et al. 2009, Mutiso et al. 2011). The six compounds tested showed in vitro inhibitory and leishmanicidal activity against the promastigote stage, although at higher concentrations compared with AmPB. This effect could also be visualised as the loss of parasite motility after 2-3 h of exposure. IC_50_ values estimated for the 2-nitrovinylfurans were comparable to those of standard antileishmanial drugs as well as of active investigational compounds in the promastigote screening system (Vermeersch et al. 2009, Hazra et al. 2012, Rocha et al. 2013).

Brominating the nitrovinylfuran scaffold either at the furan ring or at the vinyl moiety increased the electron density at C1, the double bond polarisation and, consequently, the antileishmanial activity. The methylation of C2 had a similar effect. Accordingly, the most active compound was UC245, which had the highest double bond polarisation and electron density at C1, whereas the least active was G0, with minimal electron density values and double bond polarisation.

The antibacterial mechanism of action reported for furvina, which is not necessarily the same for *Leishmania* promastigotes or for the rest of the compounds tested, is the inhibition of protein synthesis by targeting the small ribosomal subunit. In bacteria, furvina binds at or near the P-decoding site and inhibits its function, thereby interfering with the ribosomal binding of fMet-tRNA during 30S initiation complex formation and ultimately inhibiting translation (Fabbretti et al. 2012). Furvina also inhibits several key enzymes, including glyceraldehyde-3-phosphate dehydrogenase, glucose-6-phosphate dehydrogenase, malate dehydrogenase, glutathione reductase and UDP-N-acetylglucosamine enolpyruvyl transferase (Drobnica et al. 1981, Scholz et al. 2013).

Previous works suggest that these compounds react with thiol groups of proteins, among them, cysteine residues of enzymes (Sturdík et al. 1979, Scholz et al. 2013). Nucleophilic addition at the exocyclic double bond is favoured by molecular changes resulting in increased electron density at C1. Mulliken charges calculated for the vinylic carbon atoms demonstrated that increased electron density at C1 was also associated with enhanced antileishmanial activity. However, previous works demonstrated that the antimicrobial activity of nitrovinylfuran compounds may depend upon not only the parent compound, but also the degradation products and those resulting from the reaction with biomolecules (Scholz et al. 2013). Thus, rational optimisation should take into account the structural properties of the compound itself as well as of its potential by-products.

The sensitivity of KB cells to podophyllotoxin, the positive control for cytotoxicity, was comparable to previous results (Guo et al. 2011, Li et al. 2013). The test compounds were considerably less toxic than podophyllotoxin, but their cytotoxicity was still high. The lack of correlation between activity and cytotoxicity suggests that although nitrovinylfurans may inhibit enzymes shared by microbial and mammalian cells, the overall effect might depend on different biological targets, hence making molecular optimisation possible.

Two compounds were selected for in vivo assays: furvina, which is the active ingredient of Dermofural^(r)^ (BioCubaFarma, Cuba), an ointment that is licensed for the treatment of fungal cutaneous infections and thus has plenty of available chemical, preclinical and clinical information that would expedite its indication for CL if it works, and UC245, which was the least toxic according to in vitro assays and preliminary toxicological tests. The doses used in the efficacy studies were the highest tolerated by the intraperitoneal route (and the corresponding frequency of administration), causing neither mortality nor body weight loss over 10% (unpublished observations).

Animal studies using BALB/c mice, a highly susceptible inbred mouse strain (Murray et al. 1996), demonstrated the activity of the selected compounds. Two experiments were conducted in which the treatments were started rather soon after the development of nodular lesions: in the former, the compounds were administered every 24 h and, in the latter, every 12 h. In the absence of sufficient pharmacokinetic data, the second experiment was carried out as an attempt to increase the exposure of the parasite to the compounds; however, the dose of furvina had to be halved to avoid toxicity. Although the results of both assays were comparable, a superior reduction of lesion growth was achieved when the frequency of administration was increased; at 28 days of follow up, the average lesion sizes of the mice treated with furvina and UC245 with respect to the control group were 71% and 86%, respectively, when administered every 24 h, but 59% and 55% (respectively) when administered every 12 h.

Nevertheless, the dose of UC245 could be increased to 40 times that of furvina (due to its lower toxicity) and although these two compounds resulted in similar activities in vitro, no further therapeutic effect was achieved by augmenting the dose of UC245. Although there are no pharmacokinetic data to support this statement, we believe that it could likely be a consequence of faster UC245 metabolism/excretion compared with furvina.

In the third animal study, treatment began after the lesions had chronically developed. In this experiment, both the lateral and dorsoplantar diameters were measured to better estimate the lesion size because their shapes were variable; for this reason, the results were reported in millimetres squared rather than in linear millimetres. The dose of AmPB was also augmented to 5 mg/Kg administered every 48 h in an attempt to increase its efficacy in the animal model. This is the maximum dose of AmPB that we have found reported in mouse experiments (Murray et al. 1996); it is also very close to the highest nonlethal dose for female BALB/c mice administered by a single intraperitoneal injection that we have observed in our animal studies (unpublished observations). However, the clinical response to AmPB was still moderate: the lesion sizes of the mice at the end of treatment were 90% of those of untreated controls, but those treated with furvina had lesions that were 56% of the size of controls.

BALB/c mice are highly susceptible to *L. amazo-* *nensis *(Ji et al. 2002); hence, this animal model has been extensively used to test the in vivo efficacy of investigational compounds (Chan-Bacab et al. 2003, Miguel et al. 2008, García et al. 2011, Demarchi et al. 2012, Valadares et al. 2012). Although responses to the test compounds and AmPB were demonstrated, a complete clinical cure was not achieved. This partial efficacy of AmPB has been reported (Al-Abdely et al. 1999, Parra et al. 2010) and has been associated with the high multiplication rate of parasites at the site of infection (Fuchino et al. 2008).

Together, the results showed the potential of 2-nitrovinylfuran compounds as antileishmanial agents. Further structural optimisation to improve their selectivity is theoretically feasible due to their small molecular size and has previously been suggested for their use as antibacterial drugs, as well (Scholz et al. 2013). The topical use of furvina in a formulation such as that of Dermofural^(r)^ ointment could also be highly valuable for the treatment of CL due to the wide-spectrum antimicrobial activity of furvina (Blondeau et al. 1999) and the high frequency of secondary bacterial and fungal infections that complicate the healing of leishmanial skin ulcers (Isaac-Márquez & Lezama-Dávila 2003, AlSamarai & AlObaidi 2009).

The six 2-nitrovinylfurans tested were active against *Leishmania* promastigotes and the compound furvina was particularly active in the murine model of CL.
